# Preferential gene expression in the limbus of the vervet monkey

**Published:** 2008-11-10

**Authors:** Zhenhua Ding, Jun Dong, Jason Liu, Sophie X. Deng

**Affiliations:** 1 Cornea and Uveitis Division, Jules Stein Eye Institute, University of California, Los Angeles, CA; 2 Department of Human Genetics, University of California, Los Angeles, CA

## Abstract

**Purpose:**

To elucidate the unique molecular factors and biological processes that are differentially expressed in the limbal stem cell microenvironment by comparing directly to that of its immediate adjacent structures, the cornea and conjunctiva.

**Methods:**

Total RNA was isolated and amplified from the limbus, cornea, and conjunctiva. A gene expression profile of each tissue type was obtained by using microarray technique. The transcripts in which the expression level was at least twofold higher than that in the other two tissue types were identified. The expression levels of selected genes were confirmed by quantitative reverse transcription polymerase chain reaction (QRT–PCR). Protein expression of selected genes were confirmed by an immunohistochemistry study in normal human ocular tissue.

**Results:**

There were 186 preferentially overexpressed transcripts in the limbus in direct comparison to that in the cornea and conjunctiva. Many signature genes in the cornea and conjunctiva were among the preferentially expressed transcripts obtained by the microarray data. In addition, a significant number of new genes were identified, and the expression level of all nine selected genes was verified by QRT–PCR. Protein expression levels of keratin 13, tenascin c, homeodomain only protein (HOP), and TP53 apoptosis effector (PERP) were confirmed in human ocular tissues. Functional analysis of the preferentially expressed genes in the limbus reviewed that melanin metabolism and cell-cell adhesion were among the noticeable biological processes. Chromosomal distribution of the overexpressed genes in the limbus was disproportional to that of all known human genes.

**Conclusions:**

These findings may shed light on the unique molecular components and regulation of limbal stem cells and their niche.

## Introduction

Corneal epithelial stem cells have been suggested by clinical and experimental evidence to reside at the basal layer of the limbal epithelium, and they are so called “limbal stem cells” (LSCs) [1-5]. Many molecules have been proposed as markers of LSCs [6-8]. However, there is no unique marker to directly identify LSCs, and this significantly limits the localization and characterization of LSCs and their niche [9].

The concept of stem cell niche was first introduced by Schofield in 1978 [10]. The stem cell fate is governed by intrinsic and extrinsic signals. The surrounding microenvironment or niche, which consists of niche cells, soluble factors, and extracellular matrix, provides the external signals. The underlying limbal stroma including the extracellular matrix, vascular supply, and stromal cells appears to modulate the differentiation and survival of corneal epithelial cells [9,11-13]. Because LSCs have not yet been located directly, the exact spatial arrangement of LSCs and their niche cells is largely unknown. Goldberg and Bron [14] suggested that LSCs are located within the specific limbal structure, the palisades of Vogt. Papillary projections of the stroma extend upward at the basement of the limbus, and distinct invaginations of limbal epithelium into the deep stroma (limbal crypt) appear similar to the epithelial crypts of the gut where the intestinal epithelial stem cells are located [1,15,16]. The LSC niche has been proposed to reside within these deep limbal crypts [17,18].

To understand the intrinsic factors that characterize limbal stem cells, the gene expression profile of limbal epithelial cells has been compared with that of corneal epithelial cells [19,20] or of conjunctival epithelial cells [21], but no study comparing the gene expression profiles of all three types of epithelia with the underlying stroma has been reported. The goal of the present study was to identify unique genes and biological pathways that are differentially expressed in the limbal epithelium and the underlying stroma in comparison with those in the cornea and conjunctiva. The underlying stroma was included to ensure that the samples contained the deep limbal crypts and the potential components of LSC niche.

## Methods

### Tissue collection

Eye tissue of vervet monkeys (*Chlorocebus aethiops sabaeus*) was obtained from the Department of Human Genetics at the University of California, Los Angeles, CA, through the tissue-sharing protocol adherence to the ARVO Statement for the Use of Animals in Ophthalmic and Vision Research and was approved by the Institutional Animal Research Committee of the University of California, Los Angeles. The age of the animal varied from 32 to 37 months, and the diameters of the corneas were between 10 and 11 mm. The intact globe was enucleated within 1 h after euthanasia, flash-frozen on dry ice, and stored at −80 °C. The globe was placed at −5 °C to soften the tissue to allow tissue dissection. The cornea was trephined with an 8.0 mm trephine, and the epithelium along with the stroma immediately beneath it (one-third to one-half of the total stroma) was dissected. The conjunctiva with the underlying Tenon and the 1.5 to 2 mm rim of the limbal epithelium with the stroma immediately beneath it (one-third to one-half of the total stroma) were also dissected. All dissected tissues were stored at −80 °C until the RNA was extracted.

### RNA isolation

To ensure complete homogenization, a serrated homogenizer (Omni International, Marietta, GA) was used. All samples were kept on dry ice before homogenization. Each sample was bathed in a lysis buffer provided in Qiagen RNeasy mini kits (Qiagen, Valencia, CA). β-Mercaptoethanol (Invitrogen, Carlsbad, CA) was added to the lysis buffer in a 1:100 ratio. In between periods of homogenization, the blade was rinsed twice with filtered distilled deionized (ddI) water (Millipore, Billerica, MA), once with 70% ethanol, and twice in fresh Millipore ddI water. Total RNA was isolated by using the Qiagen RNeasy mini columns (Qiagen). The quantity and quality of the extracted total RNA were assessed by the NanoDrop 1000 spectrophotometer (NanoDrop, Wilmington, DE) and the 2100 Bioanalyzer (Agilent Technologies, Santa Clara, CA). Because of the sample’s low concentration, all integrity checks were run on Agilent’s RNA Pico Chips.

### Microarray analysis

A standard starting amount of total RNA (10 ng) was used for two-round transcription amplification. Synthesis for all samples was successful and provided a sufficient yield of cRNA. Vervet monkey arrays were not available, and human arrays had previously been used for other primate gene profiling without discernible loss of information. Use of human arrays for other primate gene expression studies has been very productive [22,23]. Affymetrix U133 plus 2.0 human expression arrays (Affymetrix, Santa Clara, CA) was used in accordance with the standard Affymetrix protocol for eukaryotic expression arrays. All microarrays were scanned by using an Affymetrix 3000 one-color microarray scanner. Raw images were examined for surface defects and for proper grid placement. Background intensity, housekeeping gene expression, and a 3′- to -5′ ratio of probe sets for genes of varying lengths were also used to assess the quality. Probe intensity values were generated by using the Affymetrix Gene Chip Operating System.

### Quantitative real-time polymerase chain reaction

Total unamplified RNA was used for reverse transcription. Gene-specific primers were designed (Table 1). The cDNA of each transcript was reverse-transcribed by using Superscript II RNase H2 reverse transcriptase (Invitrogen) according to the manufacturer’s recommendations, in triplicate. The relative abundance of transcripts was detected using a Stratagene Mx3000P real-time polymerase chain reaction (PCR) system with Brilliant SYBR Green QRT–PCR Master Mix (Stratagene, La Jolla, CA). Cycling conditions were as follows: an initial denaturing step of 5 min at 94 °C and subsequent 40 cycles of amplification in which each cycle consisted of 15 s at 94 °C, 30 s at 55 °C, and 30 s at 72 °C. To generate a dissociation curve after the amplification cycles, each sample was incubated at 95 °C for 1 min followed by a melting curve program (55-99 °C with a 5 s hold at each temperature). The fluorescence intensity of each sample was acquired during the execution of the melting curve program and normalized in relation to that of the housekeeping gene, glyceraldehyde-3-phosphate dehydrogenase (*GAPDH*). The average value of the triplicates from each transcript was used for comparison.

**Table 1:** Primers used for QRT–PCR.

**Gene name**	**Direction**	**Primer sequence**
*MYBPC1*	Forward	TCCAGATTGTTGACCGTCCA
	Reverse	CACTGAGGCCGCACATGTT
*TYRP1*	Forward	GGTGCAACGTCTTCCTGAACC
	Reverse	CATCAAAGACTGCATCCGTGA
*K13*	Forward	CAGAGCGTGGAGGCTGACAT
	Reverse	CCTCCTTGTTCAGCTCTGCAC
*WIF1*	Forward	CACCTGCTTTAACGGAGGGAC
	Reverse	CAGTGTCTTCCATGCCAACCT
*HPGD*	Forward	GGTGAAGGCGGCATCATT
	Reverse	GCAATCAATGGTGGGTCCA
*HOP*	Forward	ATGTCGGCGGAGACCGCGAGCGG
	Reverse	TTAGTCTGTGACGGATCTGCAC
*TNC*	Forward	GTGGAGAGCTTCCGGATTACC
	Reverse	TACTCCACTGTGTTCCCGGAC
*PERP*	Forward	CTGCTCCTACTCAGCGCCAT
	Reverse	CAAAGCCGTAGGCCCAGTTAT
*K12*	Forward	CCAGGTGAGGTCAGCGTAGAA
	Reverse	CCTCCAGGTTGCTGATGAGC
*GAPDH*	Forward	CGACCACTTTGTCAAGCTCA
	Reverse	AGGGGTCTACATGGCAACTG

Abbreviations: *MYBPC1*, myosin binding protein C; *TYRP1*, tyrosinase-related protein 1; *K13*, keratin 13; *WIF1*, Wnt inhibitory factor 1; *HPGD*, hydroxyprostaglandin dehydrogenase 15-(NAD); *HOP*, homeodomain-only protein; *TNC*, tenascin C; *PERP*, TP53 apoptosis effector;  *K12*, keratin 12; *GAPDH*, glyceraldehyde-3-phosphate dehydrogenase.

### Data analysis

All Affymetrix data were normalized by using the justRMA algorithm of R software from the Bioconductor group [24], which implements the RMA (robust multi-array average) normalization method [25]. In this normalization step, each array was individually normalized by combining it with a pool of 50 fixed reference arrays in the Microarray Core Facility at the University of California, Los Angeles. Genes with expression values at least twofold greater than those in the other two tissue types were selected and considered to be differentially expressed. EASE (expression analysis systematic explorer) software (NIH, Frederick, MD) was used for functional analysis [26]. Lists of differentially expressed genes were checked by EASE to find the most over-represented gene groups.

### Immunohistochemistry

Human sclerocorneal tissues were obtained from the San Diego Eye Bank (San Diego, CA). The death to preservation time was less than 12 h. The tissue was then cut into four quadrants and embedded in OCT on dry ice within six days from procurement. Tissues were cut into to 6 μm sections using cryostat and stored in −80 °C. Rabbit polyclonal anti-tenascin c (TNC) and anti-keratin13 (K13) antibodies were obtained from Santa Cruz Biotechnology Inc. (Santa Cruz, CA). The polyclonal rabbit anti-HOP [27] was a generous gift from Dr. Eric Olson (University of Texas Southwestern Medical Center at Dallas, Dallas, TX), and the rabbit anti-PERP antibody was from Abcam (Cambridge, MA). Frozen section slides were warmed up in the desiccator at room temperature, fixed with 4% formaldehyde for 20 min, washed with 0.3% triton in phosphate buffer saline (PBS) three times, and blocked with 5% normal goat serum (Jackson ImmunoResearch Laboratories, West Grove, PA) in PBS for 30 min. The slides were washed with 1% BSA/PBS three times, and incubated with primary antibodies overnight at 4 °C. The slides were washed with 1% BSA/PBS three times and labeled with the appropriate secondary antibodies, Alexa Fluor 488 goat anti rabbit IgG or Alexa Fluor 488 goat anti mouse IgG (Invitrogen) for 1 h at room temperature. The nucleus was labeled with Hoechst 33342 at 0.5 μg/ml for 10 min. The slides were washed with PBS three times and mounted. The pictures were taken under 25X object using a Zeiss fluorescent microscope (Carl Zeiss Inc., Oberkochen, Germany).

## Results

The RNA yield was 568.2 ng, 60 ng, and 371.4 ng from the limbus, cornea, and conjunctiva, respectively. The RNA quality assessed by using the Pico Chip revealed a flat baseline with no significant tailing of the rRNA bands (data not shown), and the S18 to S23 ratio was between 1.8 and 2.0. The RNA isolated from all three tissues appeared to have little degradation.

**Figure 1:**
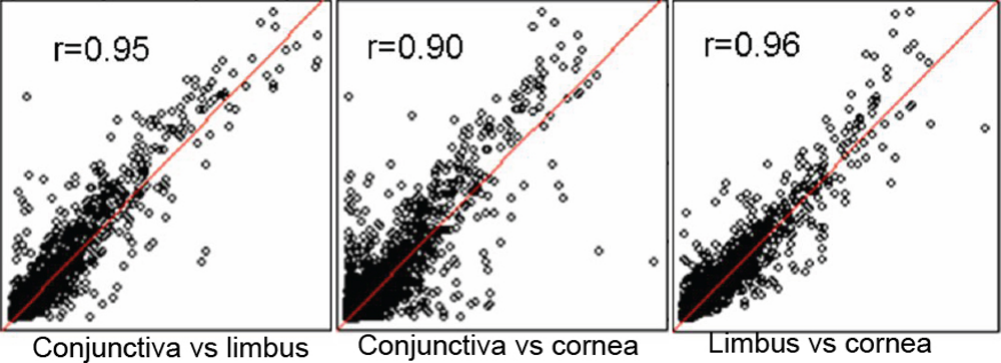
Pairwise scatter plot analysis of the correlation of all transcripts among the limbus, cornea, and conjunctiva indicated an overall similarity among all three tissue types. A slightly lower correlation was seen between the conjunctiva and cornea.

To investigate the overall gene expression profiles of the limbus, conjunctiva, and cornea, we used the Affymetrix Human U133 plus 2.0 array, which contains 54,675 probe sets representing approximately 47,000 transcripts and variants. The Affymetrix U133 plus 2.0 human expression array was chosen because of the relatedness between humans and other primates. The RMA normalized data from all three tissue types were deposited into the Gene Expression Omnibus (accession number GSE10683). The pairwise scatter plots of the normalized values indicated an overall similarity in gene expression among the three tissue types (Figure 1). A closer correlation was observed between the limbus and cornea (r=0.96) and between the limbus and conjunctiva (r=0.95) while a slightly lesser correlation was seen between the cornea and conjunctiva (r=0.90). The gene in which expression in one tissue was at least twofold higher than that in the other two tissues was considered to be differentially expressed. There were 186 transcripts predominantly overexpressed in the limbus, 644 in the cornea, and 506 in the conjunctiva. Of the 186 preferentially expressed transcripts in the limbus, 172 encoded proteins with known functions. The preferentially overexpressed genes in the limbus were ranked based on the average ratio of expression against those in the cornea and conjunctiva. The top 50 transcripts were listed in Table 2.

**Table 2:** The top 50 preferentially overexpressed transcripts in the limbus that were selected according to the ranking of the average ratio of expression level.

**Gene title**	**Gene symbol**	**Public ID**	**Average ratio**
**Cell adhesion**			
myosin binding protein C, slow type	*MYBPC1*	BF593509	23.4
Collagen, type VIII, alpha 1	*COL8A1*	AL359062	5.04
desmoglein 3 (pemphigus vulgaris antigen)	*DSG3*	AI813438	4.85
cadherin 6, type 2, K-cadherin (fetal kidney)	*CDH6*	AU151483	4.06
Protocadherin 9	*PCDH9*	R49295	3.62
tenascin C (hexabrachion)	*TNC*	NM_002160	6.55
**Developmental process**			
keratin 13	*KRT13*	NM_002274	11.04
tumor necrosis factor receptor superfamily, member 19	*TNFRSF19*	BF432648	7.72
secretogranin II (chromogranin C)	*SCG2*	NM_003469	6.02
WNT inhibitory factor 1	*WIF1*	NM_007191	5.61
v-jun sarcoma virus 17 oncogene homolog (avian)	*JUN*	NM_002228	3.9
mohawk homeobox	*MKX*	AW023227	3.67
**Neurological and nervous system development**			
coagulation factor C homolog, cochlin (Limulus polyphemus)	*COCH*	BC007230	6.43
retinol binding protein 4, plasma	*RBP4*	NM_006744	6.15
proenkephalin	*PENK*	NM_006211	5.39
proteolipid protein 1 (Pelizaeus-Merzbacher disease, spastic paraplegia 2, uncomplicated)	*PLP1*	BC002665	5.12
paired box gene 3 (Waardenburg syndrome 1)	*PAX3*	AA194168	4.31
very low density lipoprotein receptor	*VLDLR*	L22431	8.75
Netrin G1	*NTNG1*	AW051597	7.22
pleiotrophin (heparin binding growth factor 8, neurite growth-promoting factor 1)	*PTN*	AL565812	3.44
sodium channel, voltage-gated, type II, beta	*SCN2B*	AA447729	3.41
**Melanin metabolic process**			
tyrosinase-related protein 1	*TYRP1*	NM_000550	19.16
tyrosinase (oculocutaneous albinism IA)	*TYR*	BC027179	5.58
dopachrome tautomerase (dopachrome delta-isomerase, tyrosine-related protein 2)	*DCT*	NM_001922	4.27
**Transport**			
six transmembrane epithelial antigen of the prostate 1	*STEAP1*	NM_012449	8.08
solute carrier family 7 (cationic amino acid transporter, y+ system), member 2	*SLC7A2*	AA876372	6.44
solute carrier family 35, member F3	*SLC35F3*	BF968270	4.88
solute carrier family 16 (monocarboxylic acid transporters), member 6	*SLC16A6*	AI873273	4.79
potassium voltage-gated channel, shaker-related subfamily, member 2	*KCNA2*	BF513715	3.54
solute carrier family 28 (sodium-coupled nucleoside transporter), member 3	*SLC28A3*	NM_022127	3.52
**Others**			
unknown	unknown	H99792	10.3
phosphoenolpyruvate carboxykinase 1 (soluble)	PCK1	NM_002591	9.6
Transcribed locus, moderately similar to NP_775622.1 transmembrane protein 28	unknown	H09780	8.98
hydroxyprostaglandin dehydrogenase 15-(NAD)	HPGD	NM_000860	6.61
glutathione peroxidase 2 (gastrointestinal)	GPX2	NM_002083	6.6
melan-A	MLANA	U06654	5.94
roundabout, axon guidance receptor, homolog 2 (Drosophila)	ROBO2	AB046788	5.33
CAZ-associated structural protein	CAST1	Z38645	5.23
lipoma HMGIC fusion partner-like 3	LHFPL3	AI939602	5.06
Chromosome 8 open reading frame 42	C8orf42	AI632224	4.97
brain-specific angiogenesis inhibitor 3	BAI3	NM_001704	4.87
adenylate cyclase 1 (brain)	ADCY1	AL120173	4.65
hypothetical protein LOC283713	LOC283713	T03743	4.52
sushi domain containing 4	SUSD4	BC004888	3.77
L-threonine dehydrogenase	TDH	NM_152566	3.76
zinc finger protein 236	ZNF236	AA004757	3.68
family with sequence similarity 107, member B	FAM107B	BC004872	3.55
chromosome 1 open reading frame 74	C1orf74	AW295407	3.53
low density lipoprotein receptor-related protein 11	LRP11	BF696304	3.51
EPS8-like 1	EPS8L1	AF282167	3.51

The transcripts were grouped based on their function.

**Figure 2:**
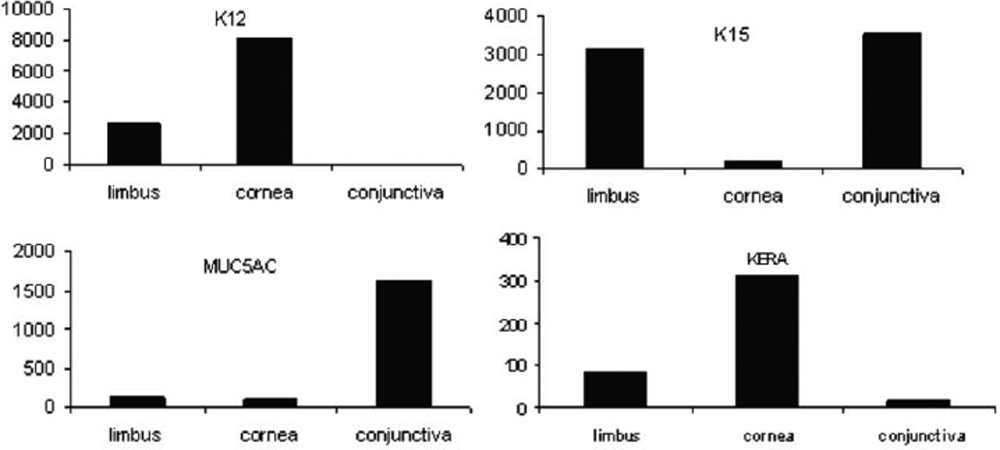
Expression levels of selected signature genes in the cornea and conjunctiva obtained by the microarray method. Abbreviations: K12, keratin 12; K15, keratin15; MUC5AC, mucin 5AC; KERA, keratocan.

To validate our microarray data, we analyzed the expression pattern of several well known signature genes in the cornea and conjunctiva. One of the cornea epithelium markers, keratin 12 [2], and the cornea stroma protein, keratocan [28], were predominantly expressed in the cornea and minimally expressed in the conjunctiva and limbus (Figure 2). Mucin 5AC, a conjunctiva marker [29], was preferentially expressed in the conjunctiva but not in the other two tissues (Figure 2). Other markers such as keratin 15, which had been shown to be exclusively expressed at the basal epithelial layer of the limbus and conjunctiva [30], had a higher transcription level in the limbus and conjunctiva (Figure 2). The expression levels of all of these signature genes of each tissue type were highly correlated with their expected expression patterns.

**Figure 3:**
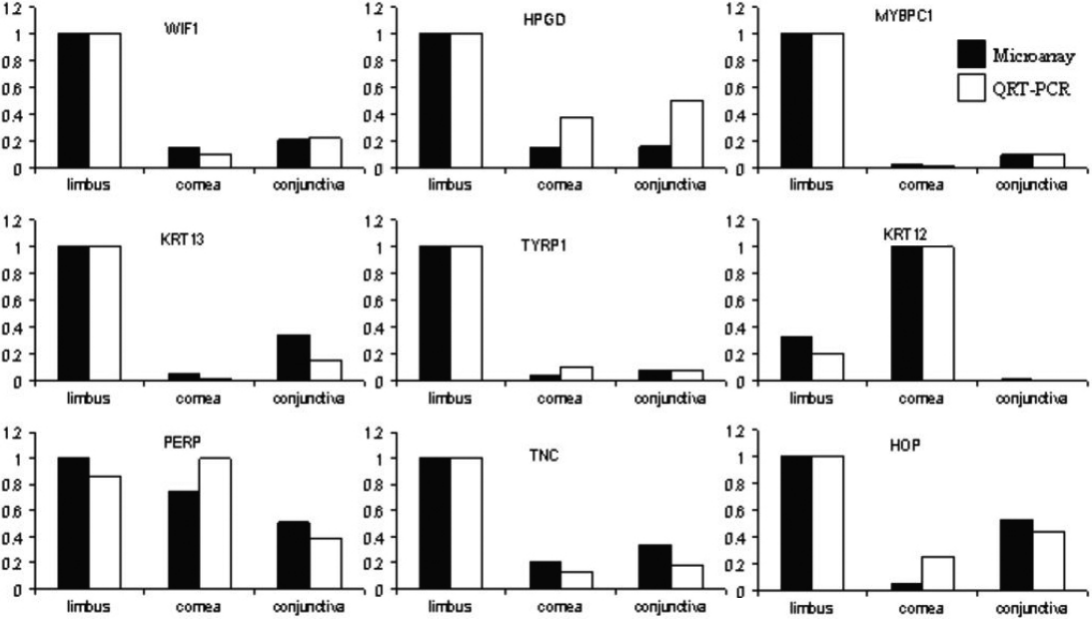
Levels of mRNA of nine transcripts obtained by microarray method were comparable to those obtained by QRT–PCR. Solid bars represent expression values obtained by microarray, and the white bars represent expression values obtained by QRT–PCR. Abbreviations: *WIF1*, Wnt inhibitory factor 1; *HPGD*, hydroxyprostaglandin dehydrogenase 15-(NAD); *MYBPC1*, myosin binding protein C; *TYRP1*, tyrosinase-related protein 1; *KRT12*, keratin 12; *KRT13*, keratin 13; *PERP*, TP53 apoptosis effector; *TNC*, tenascin C; *HOP*, homeodomain-only protein.

To further verify the microarray method, nine transcripts with differential expression patterns seen in our microarray analysis were independently quantified by QRT-PCR. These genes included Wnt inhibitory factor 1 (WIF1), hydroxyprostaglandin dehydrogenase (HPGD), K13, K12, HOP, PERP, myosin binding protein C (MYBPC1), tyrosinase-related protein 1 (TYRP1), and TNC. To allow for direct comparison of these two methods, the highest expression value of each gene obtained by each method was independently set to 1. The expression values in the other two tissue types were calibrated proportionally for each method. In all cases, the expression levels of all nine genes seen in the microarray experiment were consistent with those measured by QRT–PCR (Figure 3).

**Figure 4:**
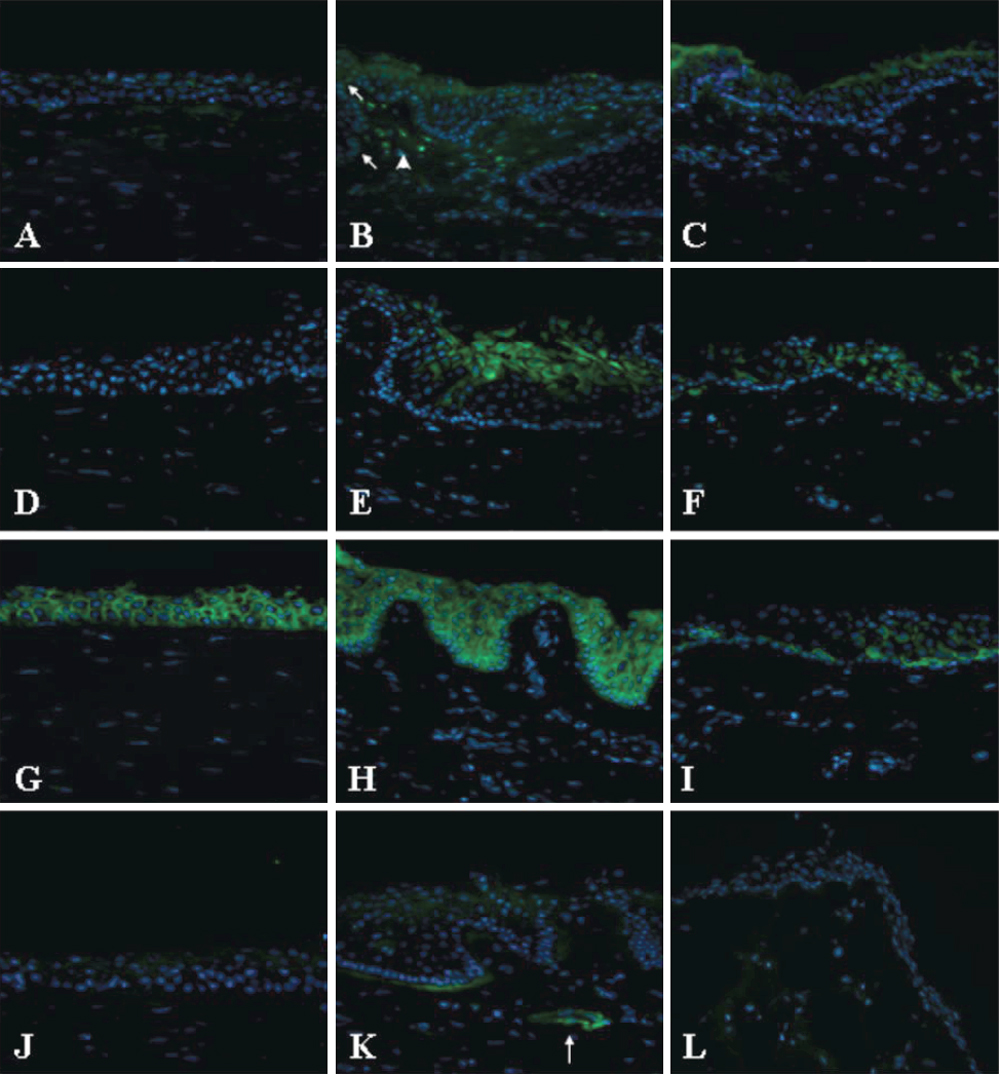
Protein expression of four selected genes in the cornea, limbus and conjunctiva by immunohistochemistry study. The left column was cornea (**A**,**D**,**G**,**F**). The middle was limbus (**B**,**E**,**H**,**K**), and the right was conjunctiva (**C**,**F**,**I**,**L**). HOP (green, A-C) was localized to the nucleus (blue) of a subgroup of basal limbal epithelial cells (**B**, arrows) and stromal cells (arrow head). K13 (green, **D**-**F**) was expressed in the suprabasal limbal (**E**) and conjunctival (**F**) epithelium and absent in the cornea (**C**). PERP (green, **G**-**I**) was localized to all layers of the epithelium in all three tissues. TNC (green, **J**-**L**) was localized specifically at the basement membrane of the limbus and blood vessel walls (arrow).

Expression of K13, TNC, PERP, and HOP at the protein level was further studied in human ocular tissues by immunohistochemistry. As showed in Figure 4, K13 was expressed at the suprabasal level of limbal and conjunctival epithelium. It was absent at the basal level in both tissues. The level of expression was higher at the limbus and totally absent in the cornea. TNC is a nonstructural extracellular matrix protein that is believed to regulate cell adhesion and migration and to have signal-altering functions [31,32]. It was expressed at the basement membrane of the basal limbal epithelium and blood vessel walls and was absent in the cornea and conjunctiva. HOP was expressed in the nucleus of a subgroup of basal epithelial cells and deep stromal cells in the limbus. It was also found in the basal layer of the conjunctival epithelium and a smaller population of stromal cells but at a much lower level than in the limbus. It was completely absent in the corneal epithelium and stroma.

EASE software was used to identify gene ontologies that were preferentially expressed in the limbus. Many important biological processes were identified. A few noticeable pathways included melanin biosynthesis and metabolism, ectoderm development, neurophysiologic processes, and cell adhesion. Melan-A, a melanocyte differentiation antigen [33], was predominantly expressed in the limbus. Melanin biosynthesis is mediated by a group of enzymes that is uniquely expressed in melanocytes and belongs to the tyrosinase-related protein (TRP) family. TYRP1 is one of the most abundant glycoproteins in melanocytic cells [34].Three members of the TRP family, tyrosinase (*TYR*), *TYRP1*, and dopachrome tautomerase (*DCT*, formally known as tyrosinase-related protein 2) [35-38], were among the top out of the 50 highly expressed genes in the limbus (Table 2).

Seven highly expressed transcripts in the limbus encode proteins that are involved in cell-cell adhesion or are components of the extracellular matrix. These proteins include TNC [39]; P-cadherin [40,41]; K-cadherin [42,43]; collagens type XI α1 [44], type VII α1 [45,46], and type VIII α1 [47]; and desmoglein [48]. All of these proteins have been implicated in the development of the cornea, the regulation of cell adhesion and migration, or the alternation of intracellular signaling.

**Figure 5:**
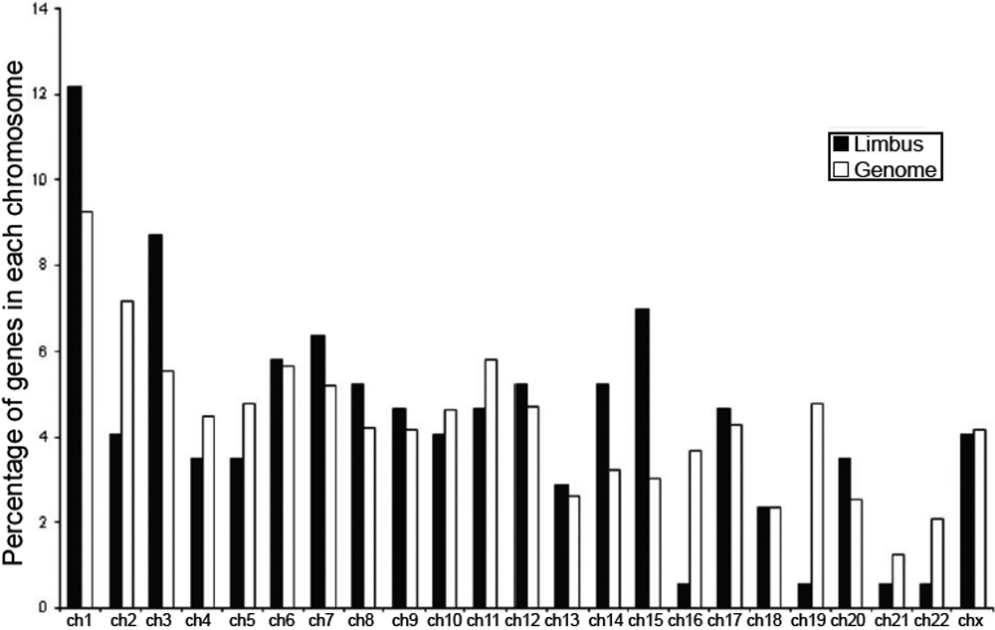
Chromosomal distribution of preferentially overexpressed genes in the limbus was disproportional to that of all other known human genes. Abbreviation: ch, chromosome.

Genes encoding human proteins were not uniformly distributed on different chromosomes, and previous studies showed that there was tissue-specific organization of genes on human chromosomes [49]. We therefore identified the chromosomal localization of the 172 transcripts of known functions that are preferentially overexpressed in the limbus and compared to the chromosomal localization of all known human genes. The analysis showed that the highest percentage of the limbus-specific genes was found on chromosome 15 when compared with that of all other known human genes (6.98% versus 3.01%). The next highest percentage was found on chromosome 14 (5.23% versus 3.23%), and the lowest percentage was found on chromosome 19 (0.58% versus 4.79%; Figure 5).

## Discussion

This study is the first to investigate the gene profile of the limbus in direct comparison with those of two adjacent structures, the cornea and conjunctiva, in the vervet monkey. The purpose of this study is to elucidate potential molecular components of the LSC niche. The epithelia and the underlying stroma are included in the analysis. The advantage of this approach allows for comparing all different cell types simultaneously to identify the unique transcript in all cell types. One limitation of the approach is that the spatial location of the transcript needs to be further verified by immunohistochemistry.

To overcome the difficulty of obtaining sufficient fresh human ocular tissues, we used ocular tissues of the vervet monkey because the monkey genome is more than 95% similar to that of the human. All three tissues types were acquired from the same eye to reduce variations seen among different animals. Many signature genes of the cornea and conjunctiva were among the preferentially expressed genes in our microarray analysis (Figure 2). In addition, the expression levels of nine selected transcripts observed in the microarray experiment were consistent with those seen in our independent QRT–PCR analysis. Protein expression of K13, HOP, PERP, and TNC in all three tissue types in humans corresponded very well to their mRNA levels detected by both microarray and QRT–PCR methods. These results further validate the microarray data.

ATP-binding cassette member 2 (*ABCG2*) and *ΔNp63α* have been proposed as potential LSC markers. Our data showed that *ABCG2* was expressed in the conjunctiva at the highest level (124) followed by the limbus (75), which was consistent with the previous finding that conjunctiva contained a higher portion of the side population than the limbus [50]. Hence, *ABCG2* was not considered specific to the limbal region according to our selection criteria. In the case of *ΔNp63α*, an isoform of *p63*, there were seven different probes on the array to identify *p63*, and the limbus had the highest expression levels detected by three of these probes but did not reach the twofold cut off level. Dr. Shigeru Kinoshita’s group [51] showed that *∆Np63α* was expressed in the basal and suprabasal layers in the epithelial cells in the limbus and conjunctiva, but at a slight lesser degree in the latter. The β isoform of the *p63* appeared to be the most specific to the basal epithelium in the limbus. To further confirm their findings, QRT–PCR using isotype specific primers would be necessary.

On the basis of the EASE annotation, we found that melanin metabolism was among the leading biological processes in the limbus in vervet monkey. This result is consistent with the observation that the limbus of the vervet monkey is highly pigmented. The palisades of Vogt, which is where LSCs are thought to reside, contain pigment granules that are aligned with the microplicae of the epithelium in human [6]. The melanocytes are scattered in the basal limbus epithelium [52]. Exposure to ultraviolet rays causes oxidative insult, DNA damage, and cell death in the corneal epithelia [53,54]. Epidermal melanocytes appear to play an important role in protecting epidermal tissue from oxidative damage. In addition, melanin pigmentation directly blocks ultraviolet radiation [55]. A recent article proposed that N-cadherin^+^ limbal melanocytes might be a cellular component of the LSC niche [8]. Whether melanocytes indeed constitute the LSC niche has yet to be confirmed. Nevertheless, there is mounting evidence that melanocytes play an important role in protecting neighboring cells from environmental insults.

Cell-cell adhesion was another highly noticeable biological process in the limbus. P-cadherin and TNC have been previously shown to be expressed preferentially in the extracellular matrix of the limbus in adults [17,20,56,57]. Cadherins play important roles in cell adhesion, motility, and development [58]. P-cadherin is most abundant in the placenta [59] and is restricted to the basal or lower layers of stratified epithelia including the prostate and skin and also to the breast myoepithelial cells [60,61]. Its aberrant expression is implicated to the cause of increased invasiveness of breast and cervical cancer [62]. TNC is another extracellular matrix protein that is only expressed at the basal membrane of the limbal epithelium (Figure 4). This finding is consistent with those of previous observations [56,57]. It is one of the nonstructural extracellular matrix proteins that are believed to regulate cell adhesion and migration and to have signal-altering functions [31,32]. More importantly, TNC is expressed in several stem niches including the nervous system [63,64], skin [65], and hematopoietic system [66,67]. In the neuronal system, TNC functions as a modulator of growth factor responsiveness in the developing neuronal stem cell niche. It also plays a director role in the retention of hematopoietic progenitor cells via interaction with stromal cells in the hematopoietic stem cell microenvironment. The specific distribution of both P-cadherin and TNC at the limbus is intriguing, and whether they play a similar role in the LSC niche is yet to be elucidated.

Our microarray data and immunohistochemistry studies showed that both K13 and HOP were preferentially expressed in the limbus, but their expression patterns were totally different. While K13 was predominantly expressed in the suprabasal epithelium, HOP was found in a subgroup of basal epithelial cells and stromal cells. Based on the expression pattern, K13 would not be a candidate of LSC marker. However, this finding indicates that the limbal suprabasal epithelia are different from those in the cornea or conjunctiva. *HOP* is an atypical homeobox gene that modulates cardiac development via cardiomyocyte differentiation and proliferation [27,68]. It interacts with serum response factor (SFR) and results in the inhibition of SRF-dependent cardiomyocyte specific genes. HOP is also expressed in the placenta and modulates the differentiation of trophoblastic cell lineage [69]. Our study is the first to identify that HOP is preferentially expressed in the basal limbal epithelial cells and the underlying stromal cells. Its unique anatomic expression suggests that it might also involve in the differentiation or proliferation of LSCs.

The chromosomal distribution of the limbus-specific genes was not random and was disproportionate to the general chromosome distribution of known human genes (Figure 5). Chromatin-remodeling factors are involved in maintaining chromatin structures and modulating gene expression in organisms ranging from yeast to human [70]. Previous studies showed that niche signals can regulate the renewing ability of stem cells at the chromatin level [71]. Whether the transcription of limbus-specific genes is regulated by chromosome-modeling factors as is the transcription of particular genes in embryonic stem cells and some adult stem cells is a very interesting idea, but its investigation is beyond the scope of the current study.

In summary, our microarray study has identified many genes that are preferentially expressed in the limbus of the vervet monkey that have not been reported previously. Expressions of HOP, TNC, K13 and PERP at the protein level are confirmed in humans. The findings of our study might provide valuable information in the molecular components and biological processes of limbal epithelial cells.
